# Computer Assisted Wargame for Military Capability-Based Planning

**DOI:** 10.3390/e22080861

**Published:** 2020-08-03

**Authors:** Jan Hodický, Dalibor Procházka, Fabian Baxa, Josef Melichar, Milan Krejčík, Petr Křížek, Petr Stodola, Jan Drozd

**Affiliations:** 1 NATO Headquarters Supreme Allied Commander Transformation, Norfolk, VA 23551, USA; jan.hodicky@act.nato.int; 2 Centre for Security and Military Strategic Studies, University of Defence, 66210 Brno, Czech Republic; dalibor.prochazka@unob.cz (D.P.); fabian.baxa@unob.cz (F.B.); josef.melichar@unob.cz (J.M.); milan.krejcik@unob.cz (M.K.); petr.krizek@unob.cz (P.K.); 3 Department of Intelligence Support, University of Defence, 66210 Brno, Czech Republic; 4 Department of Tactics, University of Defence, 66210 Brno, Czech Republic; jan.drozd@unob.cz

**Keywords:** computer assisted wargame, military capability-based planning, wargaming, capability requirements, expert system

## Abstract

Capability-based planning as an approach to defense planning is an almost infinitely complex engineered system with countless nodes and layers of interdependency, influenced by state and non-state diplomatic activities, information, military and economic actions creating secondary and third order effects. The main output of capability-based planning is the set of capability requirements needed to achieve the expected end-state. One revitalized qualitative technique that allows us to gain insights into unstructured and fuzzy problems in the military is wargaming—in its simplest form this involves manual wargaming. At the same time, there has been a push to bring computer assistance to such wargaming, especially to support umpire adjudication and move more generally towards full automation of human elements in wargames. However, computer assistance in wargaming should not be pushed, regardless of cost, towards quantitative techniques. The objective complexity of a problem often does not allow us to replicate the operational environment with the required fidelity to get credible experimental results. This paper discusses a discovery experiment aiming to verify the concept of applying a qualitative expert system within computer assisted wargaming for developing capability requirements in order to reduce umpire bias and risk associated with their decisions. The innovation here lies in applying system dynamics modelling and simulation paradigms when designing the theoretical model of capability development, which forms the core of the expert system. This new approach enables qualitative comparisons between different sets of proposed capability requirements. Moreover, the expert system allows us to reveal the effects of budget cuts on proposed capability requirement solutions, which the umpire was previously unable to articulate when comparing individual solutions by relying solely on his own knowledge. Players in the wargame validated the proposed concept and suggested how the study might be developed going forward: namely, by enabling users to define their own capabilities and not being limited by a predefined set of capabilities.

## 1. Introduction

Information gathering through data processing and its transformation into knowledge is a critical aspect in decision-making [1,2]. The current complex environment, made even more complex owing to multi-dimensional operations, diversity of weapons systems, national doctrinal discrepancies, leadership personalities, and ubiquitous information, has reinforced the need to reduce the Clausewitzian “friction” of war [3]. One means for mitigating this friction is achieved by increasing quantitative analyses to support decisions while not losing the main advantage of having human beings at the core of the decision-making process. Achieving military credibility in parallel to mathematical rigor to support decision-making processes is therefore a continuous effort. Innovation comes from knowledge discovery [4]. Thus commanders need capacity to study and re-study complex systems that create unstructured problems in order to discover innovative approaches leading to comparative advantages over an adversary [5].

Qualitative and quantitative methods to study the problem form two basic families of approaches that can benefit from each other [6]. Whether qualitative or quantitative, each method contains specific techniques to acquire information about a problem. This paper advocates the use of a qualitative approach in analyzing complex systems; military defense planning; one which builds on using an expert system in adjudicating wargaming. 

The next part describes firstly the status quo of military defense planning as the main subject of the study, and secondly elaborates on wargaming as the primary applied qualitative method of the study. This is followed by a review of the literature on quantitative methods applied in defense planning or related domains, demonstrating its current limitations. 

In their general experience of war, nations have learned that it is resource- and time-effective to prepare themselves in advance to protect their national interests. Defense planning is one way of doing this. In the early 1960s this area of planning was discussed at the US DoD when facing the challenge of finding a military forces structure to satisfy US political guidance [7]. The same objective is the task of one of the NATO main bodies, the Defense Planning Committee (DPC) [8]. From [7,8] we can conclude that the output of defense planning sets out the requirements for military forces to cover the political ambitions of the respective country or international defense organization. In the Handbook on Long Term Defense Planning we find other characteristics of defense planning, especially its long-term orientation, strategic level, and multidisciplinary character [9]. Breitenbauch and Jakobsson, as well as Stojkovic and Dahl, stress the same characteristics of defense planning [10,11]. It makes defense planning the most complex military unstructured problem to be solved. Moreover, uncertain operating environments make defense planning even more complex [12].

Leaving the Cold War era behind, NATO decided to engage more in crisis response operations and to rebuild their defense capabilities to fulfil the corresponding spectrum of military as well as non-military tasks. The practical result of this shift, embodied in the NATO Defense Planning Process (NDPP), is that output of this process is a set of capability requirements instead of list of required forces [13]. This approach is known as capability-based planning (CBP) [14].

Even though NATO nations as well as their partners have been encouraged to synchronize their processes with NDPP, i.e., to orient towards capability planning instead force planning, there are still states whose defense planning remains primarily fixated on force planning [15]. The CBP approach to defense planning offers its users a shared planning platform for formulating the necessary instrument requirements for the whole spectrum of military and non-military activities. CBP works with the generic abilities of its carriers, it provides a common platform for internal as well as external carriers, commercial or state-owned. Moreover, CBP links defense planning and the planning of operations more effectively than force planning.

NATO defines military capability as the ability to create an effect by employing an integrated set of aspects, categorized as doctrine, organization, training, materiel, leadership development, personnel, facilities, and interoperability [16]. Hinge defines military capability as the ability to achieve a desired effect in a specific operating environment [17]. Within this study a theoretical model of capability is introduced describing the changing character of a capability over time. Capability is described by its extent and effects, for example: “Capable of joint and combined expeditionary warfare and tactical deployment in extreme hot and cold weather conditions and of operations in most terrains under austere conditions.”

**Figure 1:**
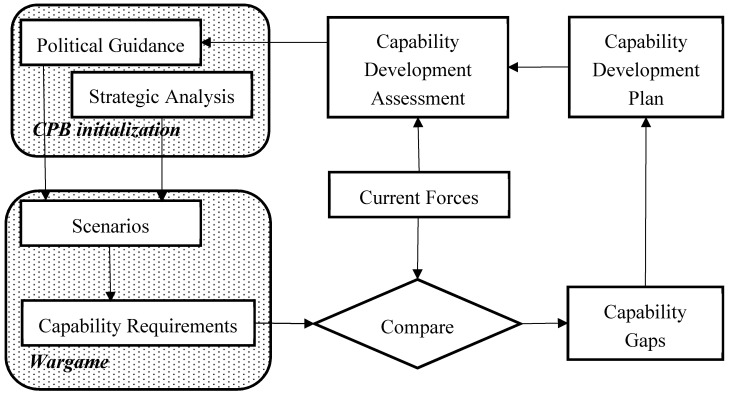
Capability-based planning cyclic process initialized by national political guidance and strategic analysis delivering a capability development plan [18].

Figure 1, originated by Hodicky and Melichar, describes an overview of the CBP process and its phasing applied at the national level [18].

National political guidance, in parallel with strategic analysis that predicts the political and military climate at a 20-year horizon, starts the CPB process and forms scenarios at the strategic level describing the future operational environment. Selected scenarios create stimuli for a wargame that generates capability requirements covering all potential threats and national political ambitions. A comparison is drawn between current forces and required capability, producing capability gaps. A capability development plan contains milestones for each capability development, driving all mid- and long-term military investment. Capability development assessment creates feedback to the CBP through updated political guidance reflecting the necessary changes in capability development [18].

Spiegeleire specifies the trends in capability-based planning, clearly articulating the need to have more insight on the defense planning process in the complex operational environment of the future [19].

Qualitative approaches for investigation involve open-ended questions and personal definitions or interpretations in order to describe or understand an event [20]. In the military sphere, one revitalized qualitative technique that enables the acquisition of insights is wargaming—in its essential form, manual wargaming. In recent years, the U.S. military and NATO has rediscovered wargames as an effective way to explore increasingly difficult problems [21]. Moreover, the use of wargames is not only limited to the military, examples of their use and value exist in different domains [22], although they lie beyond the scope of this paper.

Historically, wargames are a military staple. In the 5th century B.C., Sun Tzu introduced Wei Hai (encirclement) as a fundamental tool for understanding concepts in the Art of War. The most serious advance in wargaming occurred, however, thanks to the efforts of George Heinrich Rudolph Johann von Reisswitz, who pioneered realism in his kriegsspiel [23].

The following definitions of a wargame enable us to formulate its fundamental elements and helps us understand the study methodology.

Perla defines a wargame as: “A warfare model or simulation, using rules, data, and procedures, not involving actual military forces, and in which the flow of events is affected by, and in turn affects, decisions made during the course of those events by players representing the opposing sides [23].”

NATO (2015) defines a wargame as: “A simulation of a military operation, by whatever means, using specific rules, data, methods and procedures [24].”

Red Teaming Guide (2013) defines a wargame as: “A scenario-based warfare model in which the outcomes and the sequence of events affect, and are affected by, the decisions made by the players [25].”

Even if these definitions emphasize different elements of wargames, together they form the fundamental elements of a military wargame. The wargame (WG) scenario is the scene opener for the players. It is an exhaustive description of the scene and it contains geopolitical information about the area of operation, describing political, military, economic, social, information, and infrastructure (PMESII) factors. A WG order of battle (ORBAT) contains the capability of your own forces and its characteristics. WG maps and charts create the interface between the scenario and the players. It is an extra add-on to the scenario, immersing players into the operational environment. The WG clock is the driver of the flow of the game. It should reflect needs of the objectives and the players. The WG rules and data create boundaries for the players’ moves and the decision-making process and are enforced by the umpire during the game. WG players give the wargame its dynamic quality. They manage the planning and decision-making process. Typically, there are two teams of players—blue and red. WG analysts study the results of the wargame’s execution, develop arguments to support the umpire in his decision and imposed analytical questions. The WG umpire rules over the executed plans of the players. The umpire is the main enabler of the successful cognitive sub-phase of the wargame. He is in charge of formulating the achievements, setbacks and limitations that follow from the execution of a single wargaming cycle. For further reading on wargame elements and the wargame life cycle in the military domain, refer to the Wargaming Handbook [26]. For the detailed design of game mechanics, not specific to the military wargaming domain, refer to Adams and Dormans [27].

The aforementioned definitions of WGs have a single commonality—the use of modelling and simulation (M&S). Every WG requires the replication of the operational environment in a manner corresponding to the maximum approximation of reality. Modelling processes create model of an operational environment and the model’s behavior is scrutinized in the simulation by the execution of the model in time. Simulation stimulates the players: the wargaming environment first forces them to employ their creativity in the wargame planning phase, and second it forces them to learn in the game’s cognitive phase.

**Figure 2:**
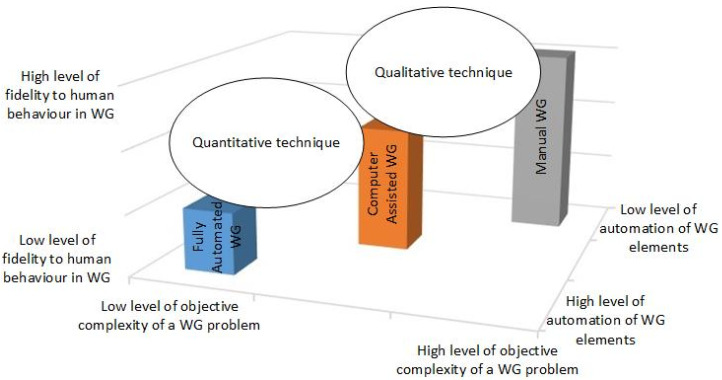
The WG classification (Manual WG, Computer Assisted WG and Fully Automated WG) in the context of the quantitative and qualitative techniques landscape [21] and its relation to the level of objective complexity of a WG problem, the level of automation of WG elements and the level of fidelity to human behavior in WG.

There is no single WG classification accepted by the WG community. The Guide for Understanding and Implementing Defense Experimentation classifies WGs based on the form of the simulation type [28]. The Red Teaming Guide manual divides wargames into two large families [25]. The first family belongs to the domain of decision support, and the second family belongs to the domain of training/education. Purnele categorizes wargames by their adjudication style: seminar (non-adjudicated), matrix, expert, and rigid [29]. The WG classification driving this study articulates the level of automation of the WG elements represented in the simulation of the operational environment [21]. Figure 2 describes the basic philosophy of the WG classification, which puts together the level of objective complexity of a WG problem (defined as the function of the number of problems/system elements/objects and their relations) [30], the level of automation of WG elements and the fidelity to human behavior represented in the WG. We created Figure 2 based on our observation of operational and strategic NATO/national WGs putting in the context of the WG classification [21] and the limitation of quantitative techniques further elaborated in the literature review in this section.

A manual WG (MWG) does not have any computer means involved in executing the WG. Human-in-the-loop simulation represents the manual WG, where the operational environment, courses of action, and effects are all fully controlled by humans. A MWG analyses an unstructured problem representing a complex system with a high level of fidelity to human behavior because it does not replace any WG human element. The fully automated WG (FAWG) has all WG human roles, like umpire, player and analyst fully automated. The closed-loop simulation with an automated analyst represents FAWGs. There is no human intervention in FAWGs, and to the authors’ best knowledge there is no such solution available. If a FAWG was implemented using state-of-the-art artificial intelligence technology, it would have low fidelity to human behavior. Computer-assisted WG (CAWG) automates specific WG human behavioral elements. As more human behavior elements are automated in CAWGs, fidelity to human behavior decreases.

Therefore, a MWG tends to be the more qualitative technique to discover information employing high levels of creativity; conversely, a FAWG represents a quantitative way to analyze a problem by a hypothesis testing. By decreasing levels of complexity, we are able to use more quantitative approaches to study a problem and vice versa.

The following examples depict the status quo when it comes to qualitative and quantitative techniques related to defense planning or related military problems.

Some examples of quantitative approaches are aimed at allocating resources to different weapons in order to effectively eliminate threat from hostile countries by optimizing single objective functions, such as effectiveness or profit. Gu at al. implemented a planning model for an armed forces structure by maximizing expected effectiveness [31]. Håkenstad at al. compared long-term defense planning systems of a selected group of countries and concluded that most countries aimed to improve their own profit [32]. Zhang et al. proposed a model that minimized the capabilities gap through a combination of weapon systems, based on given operational requirements [33]. Wan at al. introduced the constrained nonlinear optimization problem—weapon developments planning was solved by minimizing the threat caused by the enemy as the objective function [34]. The main drawback of these studies [31,32,33,34] is the lack of human behavior factors due their absence any wargame format. Therefore their applicability and credibility of results in the context of capability-based planning methods for defense planning is questionable. Moreover, the entities in the models were simplified into a form suitable for optimizing tasks, therefore the resolution of such models is very low in comparison to reality (e.g., in [33] the operational environment is simplified into the nodes of sensor, decision, influencer, and target, with a limited set of attributes).

Zhuang at al. recognize that the complexities of the wargame structure make any analytically derived solution impossible [35]. In their study of decision-making for insurgency operations, Zhuang, et al. sought to improve the development of decision-making heuristics. This effort began with an examination of behavior in a six-player MWG. The team transformed a MWG seminar game into a CAWG, with players and umpire fully automated. Automation was done through simple rules that determined how a player may implement options based on PMESII: Political (P), Military (M), Economic (E), Social (S), Infrastructure (I1), and Information (I2). The study is close to the FAWG, although no analysts were automated. The study’s limitation was that the automated players could only use the set of heuristics that the team had programmed, and the simulated operational environment did not achieve the complexity of complete defense planning—it centered around the insurgency operations, which are mostly related to the military’s operational and strategic decision-making level.

Hernandez at al. used a combination of the previous effort [35] to automate a CAWG with an automated umpire, into a CAWG with automated umpire and players, and then to apply experimentation and modified decision heuristics at specified “turns” of the CAWG [36]. The team derived their initial decision heuristics from actual HITL CAWG and verified the rules within the operational community. The study acknowledged that the heuristics would need to adapt to the dynamics of the situation and the players involved. Therefore, phased decision heuristics as stage-wise experimentation was introduced, which permitted the use of a different set of rules than the original wargame. In each turn of CAWG a new starting decision point is created and players make their decision as it was the starting point of the CAWG. This creates players’ decision trees for each CAWG turn, which are then coded and the CAWG can be examined through the specific design of experiment. The simulation Peace Support Operations Model (PSOM) that was used in the CAWG limits the study. Since it was developed as a response to socio-economic issues at the strategic level and oriented to very asymmetric operations, it therefore covers a limited spectrum of plausible military operations in defense planning.

Najgebauer at al. proposed quantitative methods designed for evaluating required capabilities, assessing existing ones, and identifying capability gaps as a reflection of scenarios identified for a country. The main limitation of the study is the verification part and the extent of plausible military operations. The discrete simulation tool used for verification is only able to verify capabilities like firing, maneuver and movement. Although such methods are therefore well implemented in current military simulations at the operational level, the rest of the effects—especially profiling at the strategic level—are not elaborated and cannot be verified. Moreover, the proposed solution is based on a closed-loop simulation, and therefore it falls into the family of CAWGs with automated players and umpire. The human element involved in finding capability requirements is missing [37].

The Joint Defense Planning Analysis and Requirements Tool Set (JDARTS) supports the NATO defense planning process. It comprises interconnected applications that drive defense planners through NATO defense planning. Two fundamental applications, the Defense Planning Mission Study Tool (D-MIST) and the Defense Planning Capability Assignment Logic Calculator (D-CALC), form the philosophical approach to NATO defense planning. D-MIST develops and stores mission types that then compose operational scenarios. The lowest level of mission decomposition is task oriented and all together they create the stimuli for D-CALC. D-CALC develops and runs scripts generating capability requirements. These scripts can take any inputs replicating implemented NATO doctrine [38]. This solution is limited because internally it does not contain any simulation that would validate the proposed capability requirements vis-à-vis the operational scenario. This can be done externally and results can be transferred into a script, however, in practice this is not feasible if you aim to cover all possible military courses of action with a high level of simulation fidelity. Usually only simple rules of thumb or static models are employed, meaning that the dynamic aspects of the capabilities life cycle cannot be implicitly reflected in this way.

Norway, as explained in [39], has already embraced JDARTS and modified it for their national defense planning process as well. Nonetheless, the previously mentioned drawback persists.

Other national efforts involve launching programs of defense planning with a defined architecture for capability-based planning, supported by various decision support tools like knowledge management, capability engineering support, concept development and experimentation, and simulation [12]. This study describes the overall framework without presenting the intricate implementation details of the proposed decision support tools.

Bychenkov at al. proposes implementing a quantitative expert system while breaking capabilities into functional groups [40]. The study describes the design of an expert system based on a single selected functional group called “engage”. This group of capabilities is relatively easy to describe through well-known quantitative parameters of a unit’s position, ammunition or military operational effectiveness when transferred to the level of capability carrier. However, the remaining functional groups, the translation of their capabilities into capabilities carriers, and the quantitative parameters are not described.

From this review, we conclude that: (1) quantitative techniques, despite their applicability, bring a high level of simplification to the operational environment; (2) MWGs should be supported by their elements automation to reach analytical rigor; (3) current CAWGs employing quantitative techniques are not able to deal with the high level of objective complexity in solving a problem without loss of credibility.

The main contribution of this paper is its verification of the concept for using a qualitative expert system to support adjudication in CAWGs for CBP as a selected approach to defense planning. The innovation here lies in applying system dynamics modelling and simulation paradigms when designing the theoretical model of capability development, which forms the core of the expert system. The system dynamics methodology facilitates the policy determination process in management of complex system behaviours over time [41]. This new approach enables a qualitative comparison between different sets of proposed capability requirements.

This paper is organized as follows: Section 2 articulates the problem statement and study constraints. Section 3 describes the methodology of a discovery experiment designed to prove the concept of CAWGs to support CBP. Section 4 sets out the study results and discussion, followed by the conclusions in Section 5.

## 2. Problem Statement

We have conducted a review of the literature on military defense planning, WGs and the quantitative methods applied in defense planning while defining the problem. We understand military defense planning as a complex system with a high level of subjective complexity. The main rationale is that CBP is an almost infinitely complex engineered system with countless nodes and layers of interdependency, dynamic and emergent behaviours, with the different types of information flow and causality in general stochastic processes, inferences and coupling structures and parameters of system dynamics [42]. It is influenced by dynamic complexity and high real-time characteristic of the current and future battlefield environment [43] characterized by non-state diplomatic activities, information, and military and economic actions that create secondary and third-order effects. Therefore, in comparison to a CAWG (with its automated umpire and players), a manual WG would be the preferable technique to apply when defining capability requirements, because it does not lose the creativity of the human elements involved in WGs (as described in Figure 2). It naturally replicates decision-making as a computational process that progressively eliminates alternatives, thereby reducing uncertainty [44].

However, the main drawback in MWGs stems from their form of adjudication, which is mostly free. In such cases the umpire makes his decision based on his best knowledge and “gut feeling” and this creates a risk to the credibility of the WG results. It contrasts to rigid adjudication where, in fact, the umpire can be automated. From our review of quantitative techniques applied in the defense domain, we conclude that they are not applicable for defense planning. Therefore, overall, we define a problem as:

“Revealing the applicability and limitations of the qualitative approach applied in the form of a qualitative expert system supporting umpire adjudication in CAWGs for CBP, especially for its role in defining capability requirements.”

We have limited our study to the moment when capability requirements are being developed based on a scenario reflecting political guidance and strategic analysis, as described in Figure 1. The remainder of the CBP process is not tackled by this study.

NATO has approved and implemented an approach to experimentation that recognizes three types of experiment [45]. The “discovery experiment” is a test to determine the efficacy of something previously untried, the “hypothesis testing experiment” is to examine the validity of a hypothesis, and the “validation experiment” seeks to demonstrate a known truth.

Following this experimentation terminology, we have designed a discovery experiment whose objective is to verify the concept of applying a qualitative expert system in CAWGs for developing capability requirements to reduce umpire bias and risk associated with their decisions.

## 3. Methodology

The methodology section contains two parts: the first describes the experiment design involving a general description of the WG design concept instantiated in Section 4 and the second elaborates in details on design and implementation of the qualitative expert system used to support the CAWG adjudication for CBP.

### 3.1. Experiment Design and Description of CBP Wargame Design Concept

The experiment design has five main phases. It starts with the WG preparation phase; the scenario scripting following the main conclusions drawn from the strategic analysis of the future operational environment. It follows the principle and structure of specific military scenario design [46] with the objective of depicting the most plausible future with details corresponding to the strategic decision-making level. The second phase in the experiment is the first round of the WG. It this case, it is a one-sided MWG with a minimum three groups of players (teams) knowing principles of CBP process, but not knowing about the experiment objective. Each team is given the same finite set of capabilities with a description of capabilities effects and the time needed to get each capability up to a state of full operational capability (FOC), where the capability is fully developed and can achieve the required effect (Time to Build FOC). Capabilities cost is not important in this round of the WG. Teams independently propose their solution for the capability requirements in the form of a list of selected capabilities needed to reach the defined end-state based on the scenario. The enemy does not create any dynamic element within the game—the scenario simply describes its capabilities and strategic objectives. Therefore, the umpire and analysts confront directly the resulting solutions with their best knowledge and expertise. They argue and explain to each team whether the expected end-state has been reached or whether key capabilities are missing from the proposed solution. The third phase, the second round of the WG, starts with the umpire introducing the teams’ first-round solution costs, the financial yearly limit for achieving the end-state given in the scenario, new information on each capability cost (FOC Cost), and each capability maintenance cost (Maintenance Cost). Teams need to reconsider their previous solution to reach the end-state while fitting into a yearly financial limit. In this round, teams’ solutions contain a list of selected capabilities and a starting time (Start Time) when each capability is to be built. In the fourth experiment phase, a qualitative expert system is used to support the umpire in its adjudication. It facilitates a qualitative comparison of teams’ solutions and their resilience to budget cuts. In the final experiment phase, teams are introduced the experiment objective and the qualitative expert system to enable the verification part of the experiment by its participants.

Our case therefore involves a CAWG in the second round of the WG, where computer assistance is represented by the qualitative expert system. This design of the discovery experiment and the wargaming design concept allows us to verify the proposed concept of using a qualitative expert system to support adjudication in CAWGs for CBP.

### 3.2. Design of the Qualitative Expert System

The expert system creating the core of the study and the means of supporting the CAWG adjudication is composed of two main parts. The first is the internal data structure of the expert system, and the second is the system dynamics capability development model.

The expert system is founded at the strategic level; it therefore demonstrates the effects of selected and developed capabilities on the operational environment through external factors. These external factors formulate a common understanding and reflect the strengths and weaknesses of the capabilities through PEMSII lenses. Ten external factors have been identified: Dependence on Foreign Military Capability, Dependence on Foreign Civilian Capability, Dependence on National Civilian Capability, Dependence on National Governmental Capability, Deterrence, Limitation (of potential enemy forces), Detection, Interoperability, Adaptability, and Escalation Character. The internal data structure of the expert system is formed as a two-dimensional array, where each capability $Cap_{i}\left(t\right)$ from the capability catalogue weight $w_{ij}$ for external factor $X_{j}\left(t\right)$ has been set as a result of a panel of experts’ technique. The weight $w_{ij}$ express the effect of $Cap_{i}\left(t\right)$ on the external factor $X_{j}\left(t\right)$ and was transformed from a qualitative expression starting from “no effect”, through “minor effect”, ”medium effect”, ”significant effect”, and “major effect” up to “eminent effect” into quantitative scale from zero up to five in order to process the model. The previously identified ten external factors correspond to the factors $X_{1}\left(t\right)-X_{10}\left(t\right)$.

Another part of the expert system internal data structure is the capability catalogue structured in the main capability categories, namely Prepare, Command and Control, Inform, Engage, Protect, Project and Sustain. The catalogue contains a limited set of capability cards, describing the attributes of these capabilities. Each capability is characterized by a capability statement explaining the capability’s effects, the resources required to build the capability *FOC Cost*, the required building time *Time to Build FOC* and the required maintenance cost *Maintenance Cost*.

The second part of the expert system is the capability development model based on a system dynamics paradigm that forms the gaming mechanics based on internal economy [27]. The purpose of the model is to evaluate and compare the solutions proposed by players forming teams for capability requirements by implementing a theoretical model of capability development. The data provided by teams is processed by a model using a system dynamics paradigm in the Ventity application. The input to the model is the capability requirements solution, a set of capability cards, created by the team. The model output provides a quantification of external factors in time. It supports a comparison and discussion of the variants of capability requirements presented by teams.

The ratio *Maintenance PCT*:

(1) $$Maintenance\;PCT=\frac{Maintenance\;Cost}{FOC\;Cost}$$

defines the required maintenance resources to maintain the achieved level of the capability *Cap*.

**Figure 3:**
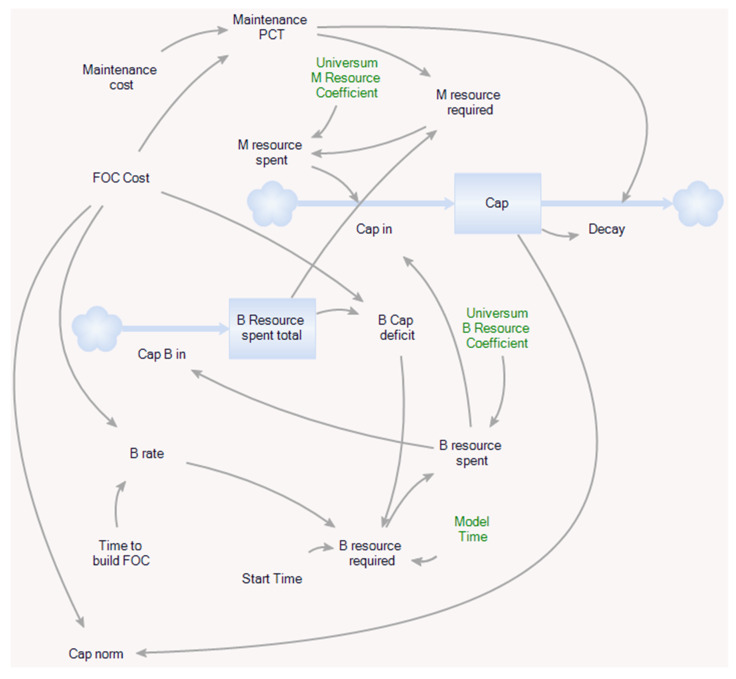
Theoretical model of a single capability described by the stock and flow diagram of a system dynamics paradigm.

The system dynamics approach is based on two basic building blocks—stock and flows. Stocks represent accumulation in the system, e.g., money on an account, while flows are variables which cause stock changes, e.g., incoming payments (inflow) or outgoing payments (outflow). Changes of a stock value is allowed only by inflows or outflows. The theoretical single capability model is represented by the scheme in the Figure 3. Stock variables are represented by blue boxes, flow variables by thick blue lines with arrows and other variables are auxiliary. The grey curved lines with arrows represent dependencies between variables.

The central variable is the *Cap* stock which represents an individual capability. The constants *FOC Cost*, *Maintenance Cost* and *Time to build FOC* determine required resources and time to build and maintain the capability. The *Cap in* inflow is money consisting of investment to build the capability *B resource spent* and money to maintain the level of capability already built *M resource spent*. If maintenance is lower than required, the capability exponentially degrades; this is represented by the *Decay* outflow. The *B* *resource required* and *M resource required* variables are the required building and maintenance resources respectively. *B* *resource required* depends on the capability *Start Time,* which is compared to the system variable *Model Time,* and on the auxiliary variable *B rate* used to determine requirement for resources per month. The *B* *resource required* variable is 0 if *Model Time < Start Time* or *B Cap deficient = 0* which means that the capability building process has not been started yet or have been finished (required building resources have been spent). The stock *B Resource spent total* with its inflow *Cap B in* accumulates building resources and it is used by the *B Cap deficit* variable to determine whether FOC has been achieved. The *Cap norm* variable express ratio *Cap/FOC* and *Model Time* is a system variable. Meaning of the *M Resource Coefficient* and *B Resource Coefficient* variables is explained below.

A capability instance is a capability card used in a solution presented by players with a specific Start Time. The value is given according to Figure 3 as:

(2) $$Cap\left(t\right)=\int_{t_{0}}^{t_{F}}\left(Cap\;in\left(\tau \right)-Decay\left(\tau \right)\right)d\tau$$

where:

(3) Decay(τ)=Cap(τ)∗Maintenance PCT

and $t_{0}$ and $t_{F}$ are the overall initial and final times of simulation.

**Figure 4:**
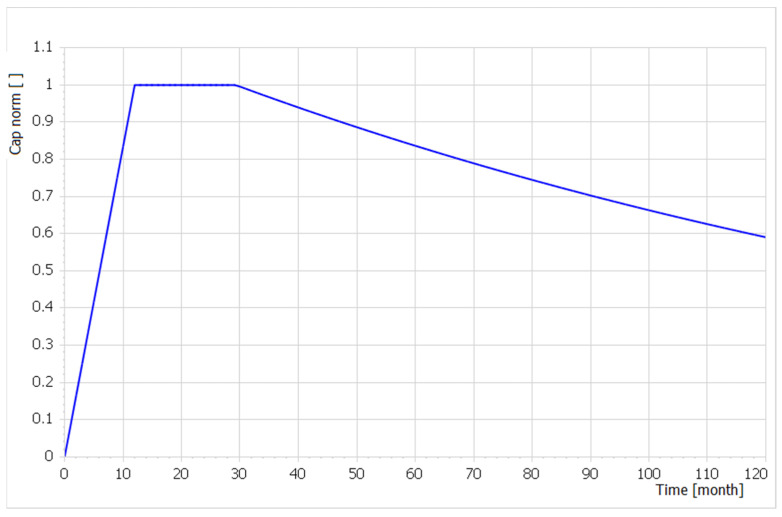
A single normed capability building, maintaining the level of FOC and its decay because of a lack of maintenance resources, expressed as the dimensionless time function *Cap norm(t)*.

Figure 4 depicts the plot of a single normed capability $Cap\;norm\left(t\right)=\frac{Cap\left(t\right)}{FOC\;Cost}$ The capability building phase took 12 months from 0 to 12, was maintained at full level *(Cap norm(t) = 1*, and a capability decay started from the 29th month due to a lack of maintenance resources.

The same capability card can be used multiple times, with the same or different start times and times to build FOC; thus, all instances of $Cap_{i}\left(t\right)$ create the set (the collection):

(4) $$\left\{Cap_{i}\left(t\right)\right\}=\overset{S_{i}}{\underset{s=1}{\sum }}Cap_{is}\left(t\right)$$

where $S_{i}$ is the number of $Cap_{i}\left(t\right)$ instances used in a particular solution. In the Excel tool, players can prolong the *Time to Build FOC* parameter for each capability instance in their solution.

If the overall resource requirement exceeds available resources, the actual resources allocated to a single capability are proportionally reduced by the coefficients *Universum B Resource Coefficient* and *Universum M Resource Coefficient*, ensuring that available resources cannot be exceeded. If resources given to players are the same or lower, there is no conflict and these coefficients are equal to 1.

External variables characterize the impact of a capability on external factors. The Universum is the space where the impacts of the capability set on external variables are calculated.

For each capability set $\left\{Cap_{i}\left(t\right)\right\}$, the impacts on the global Universum factor $X_{j}\left(t\right)$ is calculated according to the values of the capability value instances and the corresponding weight $w_{ij}$ related to the specific factor $X_{j}\left(t\right).$ The impact of a capability set $\left\{Cap_{i}\left(t\right)\right\}$ on a factor $X_{j}\left(t\right)$ is given as:

(5) $$X_{j}^{i}\left(t\right)=w_{ij}\left(\overset{S_{i}}{\underset{s=1}{\sum }}Cap_{is}\left(t\right)\right)$$

and:

(6) $$X_{j}\left(t\right)=\sum_{i}^{N}X_{j}^{i}\left(t\right),$$

where *N* is the number of different capability cards used in the evaluated solution.

For example, the impact on Deterrence caused by the capabilities of three instances of mechanized brigade (MB) and two instances of an intelligence cell (IC) is calculated as:

(7) $$X_{Deterence\;}\left(t\right)=w_{MB\;Deterrence}\left(\overset{3}{\underset{s=1}{\sum }}Cap_{MB\;s}\left(t\right)\right)+w_{IC\;Deterrence}\left(\overset{2}{\underset{u=1}{\sum }}Cap_{IC\;u}\left(t\right)\right)$$

Each instance of capability can have different *Start Time* and *Time to Build FOC* values. The other external factors are expressed the same way.

According to the scenario played, the importance of specific factors can be highlighted by the assigned weights $q_{j}$ to factors $X_{j}\left(t\right)$ and the expression:

(8) $$Global\;Impact\left(t\right)=\sum_{j=1}^{10}q_{j}X_{j}\left(t\right),\;0\leq q_{j}\leq 10$$

can be used for the overall comparison of solutions. Coefficients $q_{j}$ express the importance of external factors in respect of the scenario.

## 4. Results and Discussion

This section contains a use case of the WG preparation and execution and conclusion from the use of the qualitative expert system to support the CAWG adjudication for CBP. It describes in details who played the WG, how it was played and the way the qualitative expert system supported the second round of the WG.

General Staff course members took the role of players in the two-day discovery experiment. They are senior officers with a solid background in defense and operational planning. They were divided into three teams; each team had eight members and was supported by a facilitator who ensured that players followed the rules of the WG and were given the same level of advice. They were not inform about the main objective of the discovery experiment, i.e., to prove the concept and the existence of the qualitative expert system was not disclosed. These highly motivated teams played the WG as a part of their collective training. There was a single umpire, the former head of the Czech General Staff Defense Planning Branch, and an analytical team was composed of five retired senior officers familiar with implementation details of the qualitative expert system.

The qualitative expert system was set up before the WG; namely, a weight for each of the ten external factors (e.g., Deterrence or Detection) was set as a constant value independent of any scenario for each capability from the capability catalogue. The only scenario-dependent internal data structure consisted of weights of external factors in respect of achieving the required end-state in the scenario, expressed in the time function *Global Impact(t)*. These were set by the analytical team before the WG execution.

The umpire and their analytical team introduced the scenario to all teams together before the execution of the first round of the WG, played in the form of a MWG. The scenario was drafted as a hypothetical one, situated in 2035 in a fictitious situation after the gradual collapse of a military alliance and an economic union of countries existing on the continent. The scenario was developed to be exploited with small sized country “A” as the central player in new geo-political conditions. New geo-political conditions materialized in a regional power on the continent, with growing capabilities (military and non-military), looking to increase its influence over the continent with the ultimate goal of regional dominance. Then the umpire introduced the WG’s objective for the first day—to develop the capability requirements needed for country “A” to reach the end-state articulated in scenario, i.e., country “A” ensures its sovereignty.

Three teams developed their solution for capability requirements from the capability catalogue, which contained descriptions of ninety NATO/national existing capabilities, although their costs were concealed. This meant they planned their capability requirements without financial restrictions and produced the list of needed capabilities to reach the end-state The analytical team and umpire did not use the qualitative expert system during the first round of the WG, and they didn’t introduced any conclusion that day.

**Figure 5:**
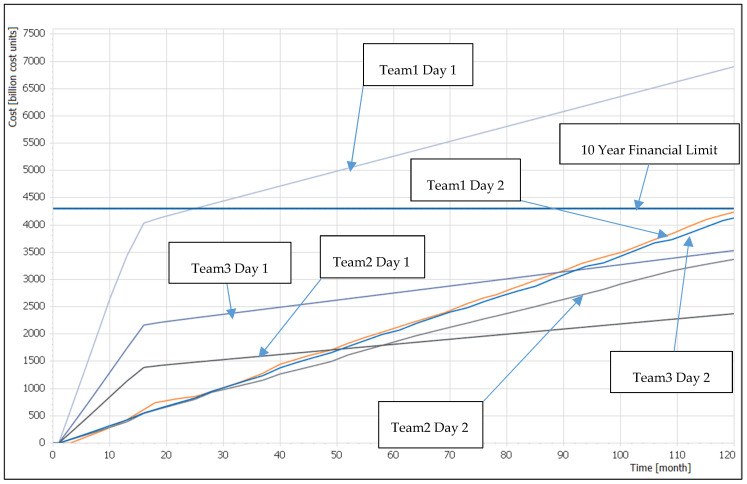
Team1, Team2 and Team3 solution cost in the first and in the second round of the WG together with the *Financial Limit* for the capability requirements introduced to teams in the second round of the WG; the solution cost is expressed as a time function putting together maintenance and capability development cost.

The CAWG was played on the second day of the experiment. As the second round of the WG, it started with the umpire stating his conclusion from the first round of the WG, solely constructed by his and the analytical team knowledge, that only Team1 achieved the required end-state and explaining the vital capabilities needed to reach the end-state. In parallel, the analytical team demonstrated the total cost of all proposed solutions from the first round of the WG, as described in Figure 5 in Day 1 legend examples. It shows that Team1, who were the only team to achieve the objective, spent twice as many resources on their first round of the WG solution than Team2 and Team3.

Then the umpire introduced the WG objective for the second day—to develop the capability requirements needed for country “A” to reach the same end-state as in the first round. However, this time a yearly financial limit was imposed on capability development and maintenance, set in the ratio of 0.5 (described in Figure 5 by the red line called Financial Limit). In this round, the capability catalogue revealed information about the cost and time to develop capabilities. Then the analytical team introduced players to the Excel tool, which would enable them to select capabilities from the capability catalogue and define their *Start Time* while controlling the *Financial Limit*. Teams were therefore forced to design their solution within the *Financial Limit*.

All teams formulated their capability requirements within the *Financial Limit*, as described in the Figure 5: this forced Team1 to curtail their first round solution and Team2 and Team3 to add capabilities in order to achieve the scenario driven end-state. The umpire and analytical team articulated the results from the CAWG by employing the qualitative expert system, and concluded the second round of the WG as follows.

**Figure 6:**
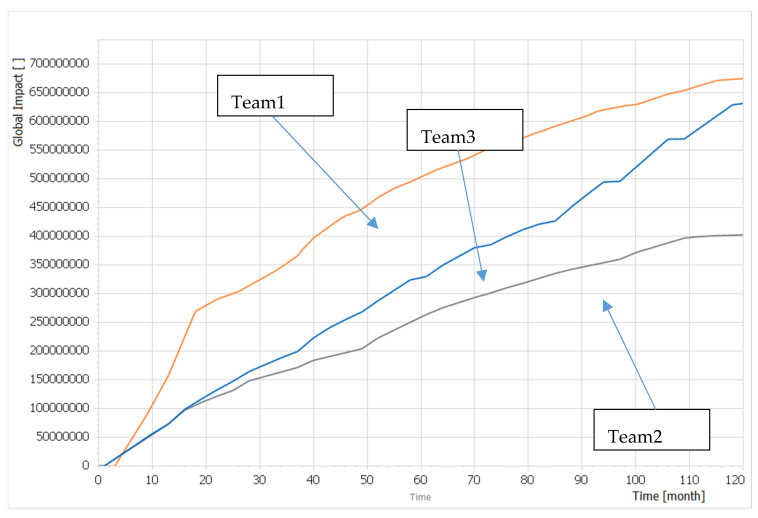
The *Global impact (t)* dimensionless time function enabling to compare Team1, Team2 and Team3 solution from the second round of the WG.

All teams were able to compose their capabilities to reach the end-state written in the scenario. Figure 6 describes the time functions *Global Impact (t)* corresponding to each team solution from the second round of the WG. Team1 and Team3 created significantly better solutions than Team2, as demonstrated by the value of the *Global Impact* at time = 120 months. This corresponds to the fact that these teams were able to spend almost completely the given *Financial Limit*. Team1 created a slightly better solution than Team3, not only because of the higher value of *Global Impact* at time = 120 months, but mainly because they were able to increase the global impact more quickly at the beginning of planning cycle; at time = 20 months, Team1 had already generated significantly greater effects with their solution than Team3.

The most important moment—clearly demonstrating the value of our approach—happened during the conclusion session of the second round of the WG, when the analytical team made changes in the qualitative expert system to the *Financial Limit*: in time = 60 months they reduced maintenance resources to the same value of 20 billion of cost units for all Teams. This reflects a very common situation in all MoDs, i.e., budget cuts introduced during the long-term planning cycle. The effects of that financial cut on the second round of the WG solutions expressed as the *Global Impact(t)* is described in Figure 8. The Team1 solution occupied a superior position over the others until time = 160 months. From that moment on, the Team3 solution generated slightly better effects than Team1′s.

**Figure 7:**
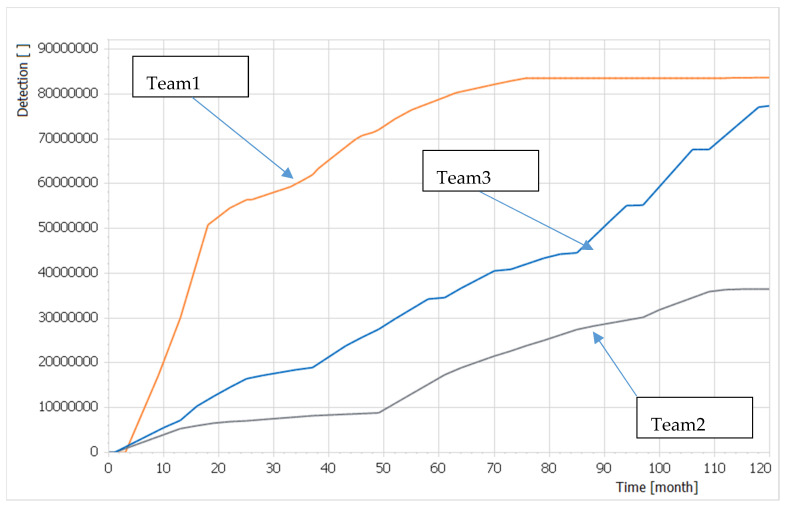
The effects of selected capabilities from the second round of the WG on the operational environment expressed by the *Detection(t)* time function as one of ten external factors in the qualitative expert system.

The qualitative expert system expresses the functions of ten external factors; as an example, Figure 7 demonstrates the time function of *Detection (t)* corresponding to each team solution from the second round of the WG. This importing finding, confirming the superiority of the Team1 solution, is reaching the maximum level of Detection even before the end of planning cycle. It corresponds to the situation in which all Detection-related capabilities are already in the FOC.

**Figure 8:**
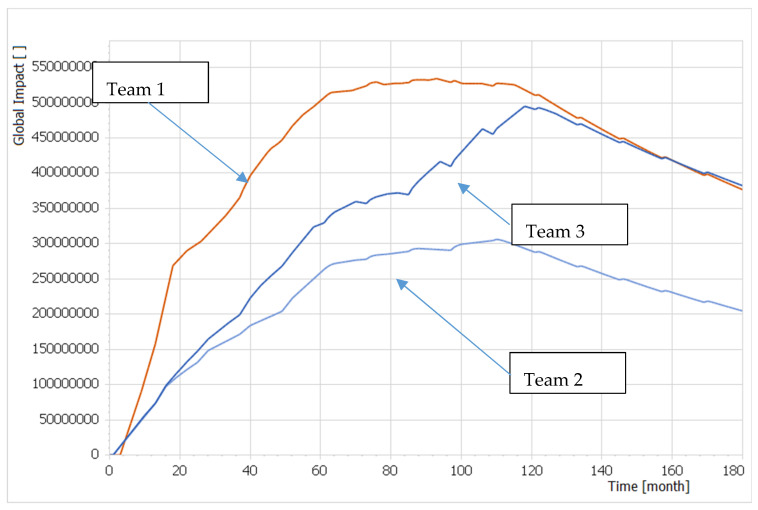
The effects of the financial cut on the second round of the WG Team1, Team2 and Team3 solution expressed as the *Global Impact(t)* dimensionless time function.

However, the finding described in Figure 8 reflects reality. If defense planners are proposing capabilities that are not necessarily needed and there is a budget cut, it is not possible to maintain already developed capabilities and overall operational effectiveness decreases. Team3′s solution was therefore more resilient to budget cuts than the others.

## 5. Conclusions

In its essence, every MWG is biased because of the human decisions reflected in the WG dynamics. Nonetheless, players’ bias is desirable: it enables the involvement of an individual’s creativity in the proposed solutions. On the other hand, umpire bias increases the risk of the WG results becoming less credible, especially when they are used for experimentation purposes and not for training or education. Concurrently, there is a constant drive within the wargaming domain to automate the human elements of WGs by quantitative approaches, thereby bringing the analytical rigor that customers need. However, the defense planning process, in our case represented by developing capability requirements, is a complex system and does not allow a move towards FAWGs, i.e., achieving full automation and validation of players and umpire. We have therefore designed a qualitative expert system as a part of CAWGs, not replicating the umpire per se, but reducing his bias and the risk associated with his decisions.

The innovative advance of this proposal lies in applying system dynamics modelling and simulation paradigm when designing a theoretical model of capability development. It allows us to reveal the effects of budget cuts, which the umpire was previously unable to articulate when comparing individual solutions for capability requirements, as described in Figure 8.

Even though the internal data structure is working with the quantitative values and outputs of the model like the *Global impact (t)*, and the values of external factors, e.g., *Detection (t)*, are also expressed in quantum, the expert system remains essentially qualitative, because its very core—weighting the effects of a capability on each external factor—has been created in qualitative way by the panel of experts’ technique. CAWGs should not be driven, regardless of cost, towards quantitative techniques. The objective complexity of a problem does not allow the operational environment to be replicated with the required fidelity to gain credible experimental results.

Participants in the discovery experiment and the WG players validated the concept of supporting comparisons between capability requirement solutions using a qualitative expert system, and also appreciated the educative dimension of the concept, which was a secondary effect of the study. Players proposed possible ways in which the study might be developed in future: namely, by extending the concept to the whole capability-based planning process and enabling users to define their own capabilities and not being limited by the current capability catalogue.
